# A novel EMT-related risk score model for Uveal melanoma based on ZNF667-AS1 and AP005121.1

**DOI:** 10.7150/jca.101823

**Published:** 2025-01-01

**Authors:** Fan Yang, Yue Ren, Dongmei Qi, Xi Liu, Jing Xie

**Affiliations:** 1 Department of Ophthalmology, Southwest Hospital, The Third Military Medical University (Army Medical University), Chongqing, P.R. China.; 2 Key Lab of Visual Damage and Regeneration & Restoration of Chongqing, Chongqing, P.R. China.

**Keywords:** uveal melanoma, lncRNA, EMT, risk score model, prognosis

## Abstract

Uveal melanoma (UM) has emerged as one of the most common primary intraocular malignant tumors worldwide. Long non-coding RNAs (lncRNAs) are increasingly recognized as decisive factors in the progression and metastasis of UM, involving in epithelial-mesenchymal transition (EMT) of UM. We conducted a comprehensive analysis of lncRNAs closely associated with EMT-related genes in the TCGA UM cohort, identifying 961 EMT-related lncRNAs. Through univariate COX analysis, we identified 9 survival-related EMT-related lncRNAs (sER-lncRNAs), further establishing an EMT-related risk scoring model (ER-RSM) with two sER-lncRNAs (ZNF667-AS1 and AP005121.1) identified by multivariate COX analysis. Through this ER-RSM, low-risk UM patients achieved better overall survival than high-risk UM patients. AP005121.1 was positively correlated with higher stage and M staging in UM patients, while ZNF667-AS1 was positively correlated with earlier stage, T, and M staging in UM patients. *In vitro*, AP005121.1 expression was higher in UM tumor tissues and cell lines than in adjacent normal tissues and human retinal pigment epithelial cells, whereas ZNF667-AS1 expression showed the opposite pattern. siR-AP005121.1 significantly inhibited migration and invasion ability of UM cells and suppressed the EMT pathway, while siR-ZNF667-AS1 promoted migration and invasion of UM cells and activated the EMT pathway. In this study, we screened sER-lncRNAs and constructed an ER-RSM to investigate the relationship between sER-lncRNAs and prognosis and clinical staging of UM. Additionally, we validated the expression of sER-lncRNAs in UM clinical samples and cell lines. The ER-RSM may provide potential key insights for the diagnosis and therapeutic intervention of UM patients.

## Introduction

Uveal melanoma (UM) is the most common primary intraocular malignancy in adults worldwide^1^. It is a malignant tumor of melanocytes occurring within the uveal tract, with 85-90% of cases involving the choroid and the remaining 10-15% occurring in the ciliary body and iris^2^. Nearly 50% of patients will experience metastasis, predominantly to the liver^3^. Primary UM are most commonly treated through radiotherapy, local excision, or enucleation. Enucleation is typically reserved for cases unsuitable for radiotherapy due to factors such as large tumor size, significant vision loss, pain, evidence of scleral invasion, severe ocular complications, or adverse events from previous eye-preserving treatments^4^. The prognosis for metastatic UM remains poor, with a median survival time of less than one year for the majority of cases^5^. Hence, there is an urgent need to investigate the underlying mechanisms of UM development and metastasis.

Metastasis is the leading cause of cancer-related deaths, especially in UM^6^. Epithelial-mesenchymal transition (EMT) is a hallmark of cancer and a significant mechanism in UM metastasis^7^. As cell-cell connections formed by E-cadherin dissolve, tumor epithelial cells lose contact with each other, become motile, and spread to adjacent and distant tissues. EMT is an evolutionarily conserved cellular plasticity program that allows polarized, immobile epithelial cells to loosen their intercellular adhesion, separate from neighboring cells, and transform into motile mesenchymal cells^8^. Various extracellular signals, such as transforming growth factor, fibroblast growth factor, hepatocyte growth factor, epidermal growth factor, and chemokines, can trigger the occurrence of EMT^9^. Once the EMT program initiates, there are significant changes in the expression of EMT-related genes^10^. These EMT-related genes ultimately have profound effects on cell morphology and function^11^. Laura has reported an EMT-associated factor promoting invasive properties of uveal melanoma cells^12^. Some researchers established an EMT-related gene signature predicts the prognosis in UM patients^13^. However, existing research has mostly focused on the coding genes induced by EMT, with relatively fewer reports on the biological functions of EMT-related lncRNAs in UM.

Long non-coding RNAs (lncRNAs) are RNA molecules exceeding 200 nucleotides in length without protein-coding potential, garnering increasing attention due to their critical roles in the occurrence, progression, and metastasis of cancer^14^. Simultaneously, lncRNAs play pivotal roles in regulating the EMT process^15^. Zhu *et al.* found that the lncRNA MIR200CHG inhibits EMT in gastric cancer by stabilizing miR-200c, which targets EMT-related factors^16^. Hu *et al.* reported that HDAC2 suppresses EMT-mediated cancer metastasis in colorectal cancer by downregulating the long non-coding RNA H19^17^. Moreover, the potential of lncRNAs in predicting patient prognosis and clinical staging is gradually being recognized. Feng *et al.* identified m6A-immune-related lncRNA prognostic features for predicting the immune landscape and prognosis of bladder cancer^18^. Ma *et al.* also discovered N6-methyladenosine-related lncRNA prognostic features for bladder cancer^19^. However, as of now, no predictive model has been constructed for UM prognosis and clinical staging based on EMT-related lncRNAs, thus necessitating the urgent development of a precise predictive model for UM prognosis and clinical staging based on EMT-related lncRNAs.

In our study, we identify two EMT-related lncRNAs (ZNF667-AS1 and AP005121.1) in UM. Following that, based on their expression, construct an EMT-related risk score model (ER-RSM) for predicting the prognosis and clinical characteristics of UM patients. We further valid that the expression of ZNF667-AS1 decreases in UM cell lines and UM tissues, while AP005121.1 exhibits opposite results. The silencing of ZNF667-AS1 significantly facilitates migration, invasion and EMT pathway of UM cell, while silencing of AP005121.1 remarkably inhibits migration, invasion and EMT pathway of UM cell. Our conclusion will provide precise prognostic biomarkers for prognostic prediction and potential therapeutic targets for target treatment of UM.

## Methods

### Cell culture and transfection

The human UM cell lines C918 and MUM-2B were cultured in RPMI1640 medium supplemented with 10% fetal bovine serum (Gibco, USA) and 1% penicillin/streptomycin (Gibco, USA). The UM cell lines OCM-1A and M619 were cultured in DMEM medium supplemented with 10% fetal bovine serum and 1% penicillin/streptomycin. The human retinal pigment epithelial cell line ARPE-19 was cultured in DMEM/F12 medium supplemented with 10% fetal bovine serum and 1% penicillin/streptomycin. All cells were maintained at 37°C in a humidified atmosphere containing 5% CO2.

MUM-2B cells in logarithmic growth phase were immediately trypsinized and seeded into 6-well plates. After 12 hours, cells reaching 60-80% confluency were transfected using Lipofectamine 2000 (Invitrogen, USA) according to the manufacturer's instructions. After 48 hours, cells were collected for further analysis. The siRNA sequences utilized are listed in Supplementary Table 1.

### Clinical samples

Ten specimens of UM and adjacent normal tissues, surgically excised at our institution from October 2022 to April 2024, were collected. All specimens were rinsed with saline and placed in Eppendorf tubes, then stored in liquid nitrogen. In accordance with the Helsinki Declaration, written informed consent was obtained from all ten participants with UM. These procedures were approved by the Medical Ethics Committee of Southwest Hospital.

### Cell migration and invasion assay

The invasive and migratory capabilities of MUM-2B cells in different treatment groups were evaluated using Transwell chambers with 8 μm pores (Corning, USA). Chambers used for invasion assays were pre-coated with Matrigel (BD Biosciences, USA). After 48 hours transfection, 3×10^4^ UM cells were added to the upper chamber of each well containing serum-free culture medium, while 700 μl of culture medium with 10% FBS was added to the lower chamber. Following further incubation for 24 hours (migration) and 48 hours (invasion), non-invading or non-migrating cells were removed from the upper chamber using a cotton swab. UM cells that invaded or migrated into the lower chamber were fixed in paraformaldehyde and stained with 0.1% crystal violet. Finally, cell counting analysis was performed by using Image J software.

### Data source

The clinical information and lncRNAs transcription data for UM were downloaded from TCGA database. The TCGA-UM dataset comprises expression data obtained from 80 tumor tissue samples. Perl script was used to integrate the downloaded data for future reference. EMT-related genes (ERGs) were obtained from MSigDB (M42501, M42502, M42503, and M42504). The status of immune cell infiltration was downloaded from the TIMER database.

### Selection of potential EMT-related lncRNAs

We conducted Pearson correlation analysis to assess the correlation between UM lncRNAs and ERGs. IncRNAs meeting the selection criteria (r>0.7, p<0.001) were considered as EMT-related lncRNAs (ER-lncRNAs).

### Construction of EMT-related risk score model (ER-RSM)

Survival-related ER-lncRNAs (sER-lncRNAs) were selected through univariate Cox regression analysis (p < 0.001). The ER-RSM was constructed using multivariate Cox regression analysis (p<0.05). The ER-RSM was created based on the sER-lncRNAs expression data multiplied by the Cox regression coefficients, and the risk score formula was as follows: [Expression levels of ZNF667-AS1 * (-1.396692192)] + [Expression levels of AP005121.1 * (0.443550867)]. UM patients were divided into the high-risk group and the low-risk group based on the median risk score of the ER-RSM.

### Bioinformatics analysis

We performed survival analysis of ER-RSM and ER-lncRNAs using the "survival" package in R. Utilize univariate and multivariate Cox regression analysis to explore whether ER-RSM and clinical indicators can serve as independent prognostic factors for predicting UM prognosis. Plot ROC curves and calculate the AUC values using the "survival ROC" package in R. The “ggplot2” package of R software was used to analyze the relations between sER-lncRNAs and UM clinical characteristics. Nomogram and calibration curves using the "rms" package in R. Kyoto Encyclopedia of Genes and Genomes (KEGG) pathway analysis was conducted using Gene Set Enrichment Analysis (GSEA, version 4.1.0).

### RNA isolation and RT-qPCR

UM tissues and cellular total RNA were extracted using TRIzol reagent (Invitrogen, USA). RNA was reverse transcribed into cDNA using the PrimeScript™ RT kit (Takara, Japan). RT-qPCR was performed on an ABI-7500 RT-qPCR system (Applied Biosystems, USA) using TB Green Premix Ex Taq™ II (Takara, Japan). β-actin was used as an internal control. Relative gene expression was presented as fold changes and calculated using the 2^-ΔΔCt^ method. Primer sequences in this study are listed in Table 1.

### Western blot

The UM cells were lysed in RIPA buffer (Beyotime, China) supplemented with 1% PMSF (Beyotime, China). Total protein content was determined using the BCA assay (Beyotime, China). Subsequently, 30 μg of protein samples were electrophoresed on a 10% SDS-PAGE gel and then transferred onto a PVDF membrane using a wet transfer system (Bio-Rad, USA). The PVDF membrane was blocked with 5% skim milk for 1 hour at room temperature, followed by overnight incubation at 4°C with appropriately diluted primary antibodies: E-cadherin (1:5,000; Proteintech, China), N-cadherin (1:5,000; Proteintech, China), Vimentin (1:5,000; Proteintech, China), and β-actin (1:5,000; Proteintech, China). After washing with TBST, the membrane was incubated with corresponding HRP-conjugated secondary antibodies (1:10,000; Proteintech, China) for 1 hour. Protein bands were visualized using an enhanced chemiluminescence method and imaged with the VILBER FUSION FX5 imaging system. Band intensities were quantified using Image J software.

### Statistical analysis

The data are presented as the mean ± standard deviation (SD) of at least three independent experiments. Data analysis, graph generation, and statistical analysis were performed using GraphPad Prism 8 software (GraphPad Software, USA). Inter-group differences were assessed using Student's t-test or one-way analysis of variance (ANOVA). A p-value less than 0.05 was considered statistically significant.

## Results

### Acquisition of sER-lncRNAs

**Figure 1:**
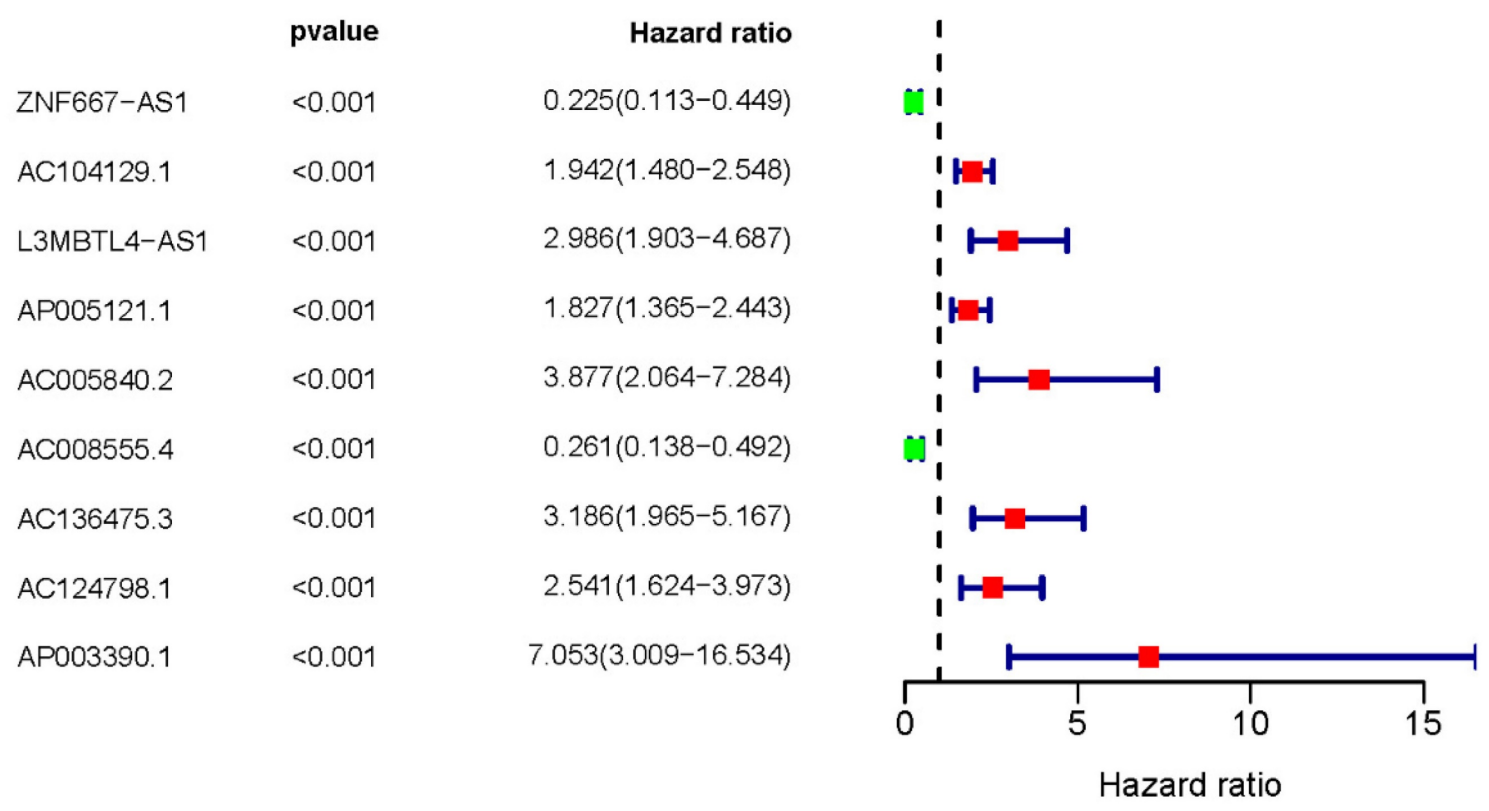
** Survival-related ER-lncRNAs.** The forest plot illustrates the UM prognostic significance of sER-lncRNAs (ZNF667-AS1, AC104129.1, L3MBTL4-AS1, AP005121.1, AC005840.2, AC008555.4, AC136475.3, AC124798.1, and AP003390.1).

We initially downloaded ERGs from the MSigDB database and lncRNA transcription data of UM patients from the TCGA database. To identify ER-lncRNAs, we employed Pearson correlation analysis to explore the correlation between lncRNAs and ERGs. In total, we identified 961 ER-lncRNAs. For further investigation into the relationship between ER-lncRNAs and the overall survival (OS) of UM patients, we conducted univariate Cox analysis and identified a total of 9 sER-lncRNAs (ZNF667-AS1, AC104129.1, L3MBTL4-AS1, AP005121.1, AC005840.2, AC008555.4, AC136475.3, AC124798.1, and AP003390.1). We then plotted a forest plot to display their HR values (Figure 1).

### Construction of ER-RSM

**Figure 2:**
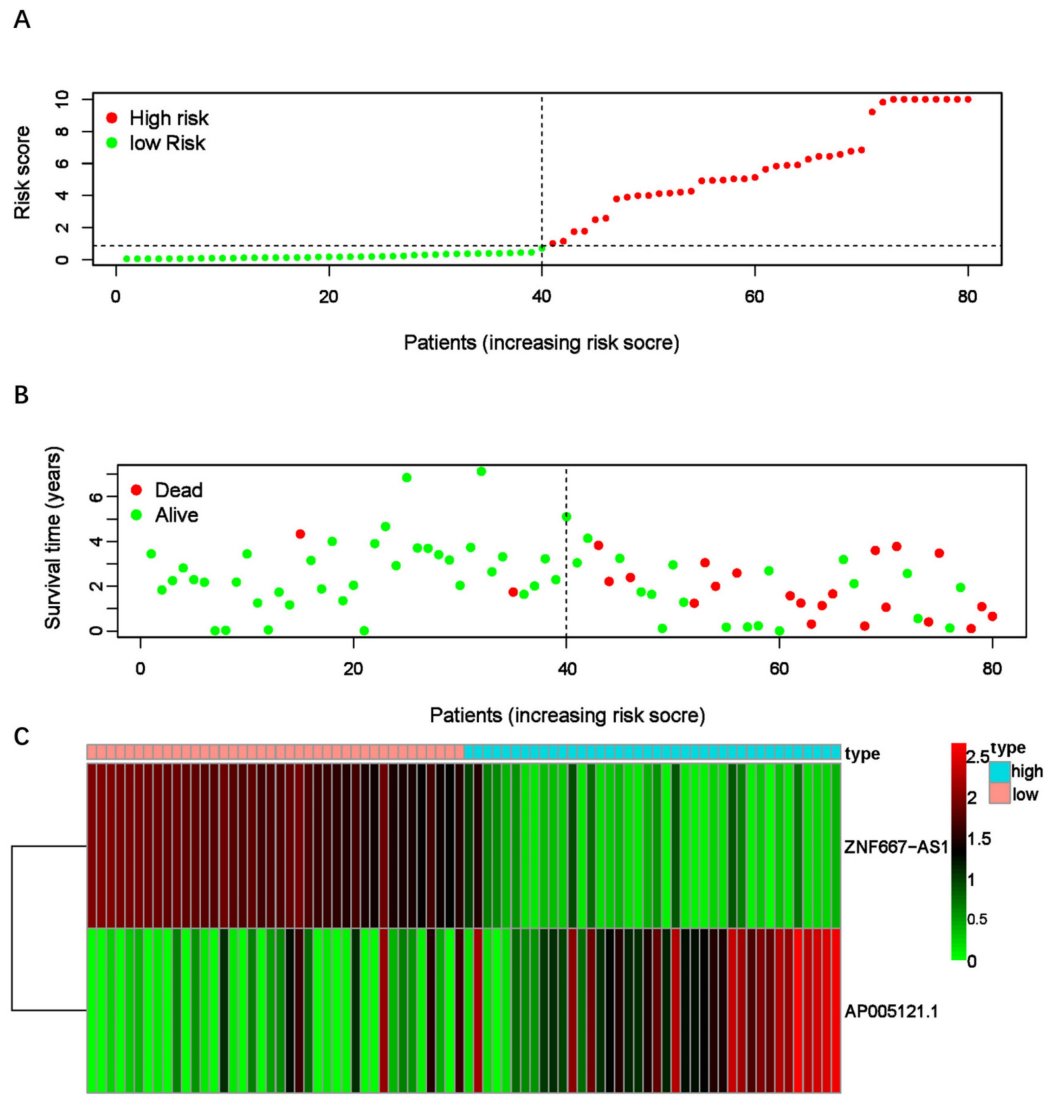
** EMT-related risk score model (ER-RSM) is constructed by ZNF667-AS1 and AP005121.1.** (A) The distribution of risk scores in the high-risk and low-risk groups of UM patients. (B) The survival status comparison between the high-risk group and low-risk group of UM patients. (C) The heatmap demonstrates the expression levels of the included sER-lncRNAs.

After confirming the sER-lncRNAs, we constructed the ER-RSM through multivariate Cox analysis. Eventually, ZNF667-AS1 and AP005121.1 were included in the risk model construction, with the risk score calculated as follows: Risk score = (-1.39669219191423 × ZNF667-AS1) + (0.443550866870963 × AP005121.1). Patients were divided into high-risk and low-risk groups based on the median risk score (Figure 2A). The high-risk group exhibited significantly more deaths than the low-risk group (Figure 2B). Compared to the low-risk group, AP005121.1 was significantly upregulated in the high-risk group, while ZNF667-AS1 was downregulated (Figure 2C).

### Predictive appliance of clinical prognosis in ER-RSM

**Figure 3:**
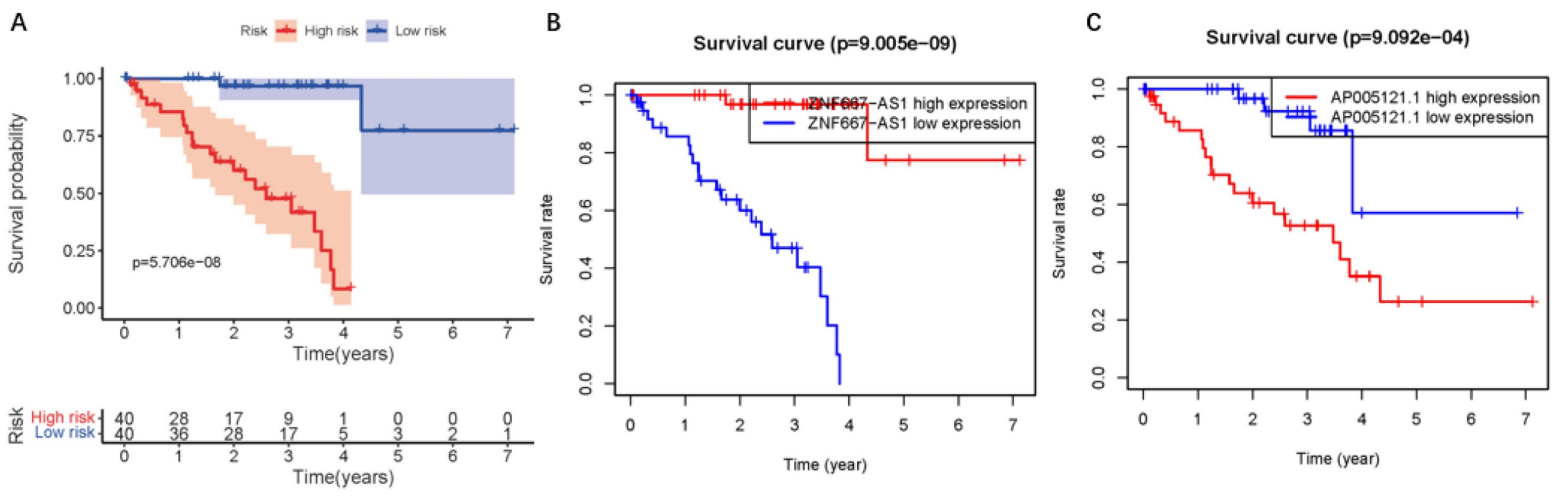
** The UM survival curve of ER-RSM.** (A) Kaplan-Meier survival curve of survival probability in the high-risk and low-risk groups of UM patients. The survival probability of ZNF667-AS1 (B) and AP005121.1 (C).

**Figure 4:**
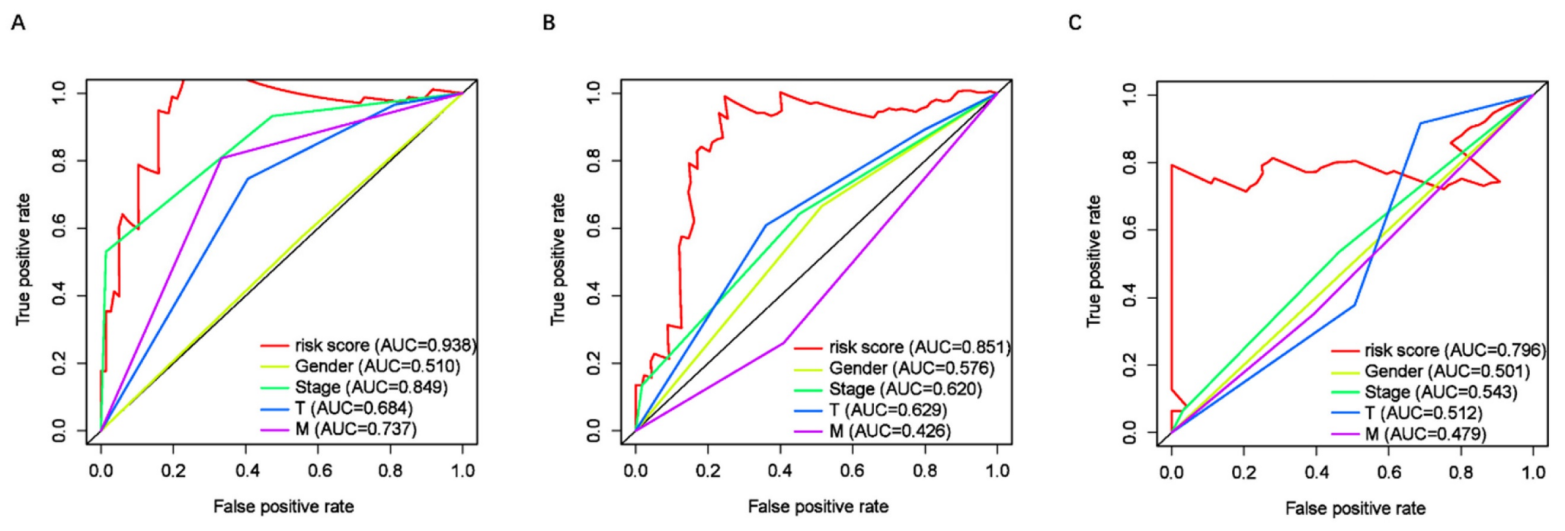
** Receiver operating characteristic (ROC) curve of ER-RSM and clinical characteristics.** ROC curves demonstrate the prognostic accuracy of risk scores, gender, stage, T-stage and M-stage in 1- (A), 3- (B) and 5- (C) year.

**Figure 5:**
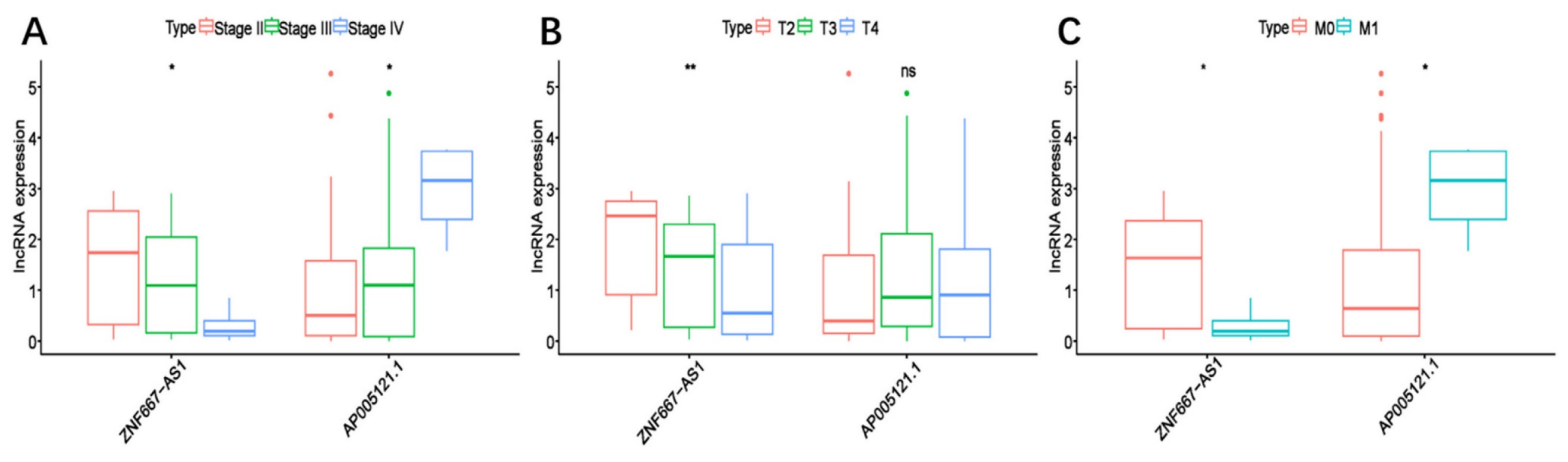
** The correlations between the ER-RSM and clinical characteristics.** Relationships between ER-RSM and stage (A), T-stage (B) and M-stage (C).

**Figure 6:**
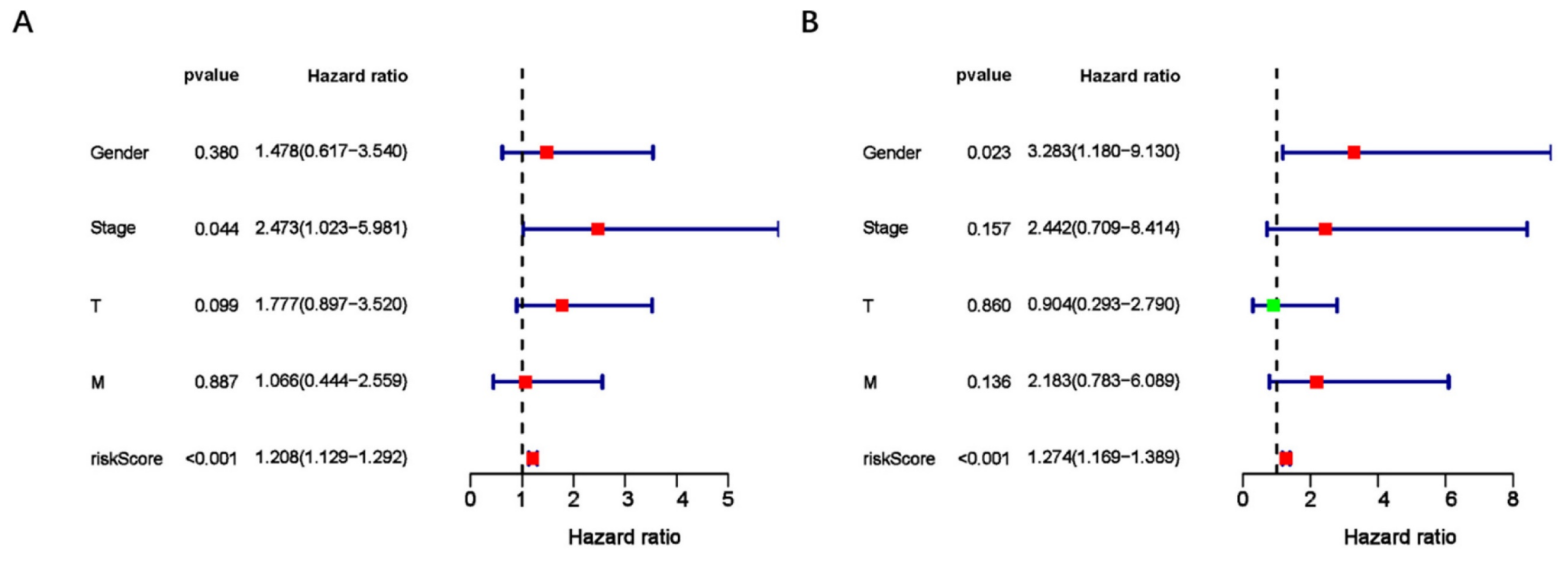
** The independent risk factor of UM patients.** Univariate (A) and multivariate (B) analysis of ER-RSM and clinical characteristics.

**Figure 7:**
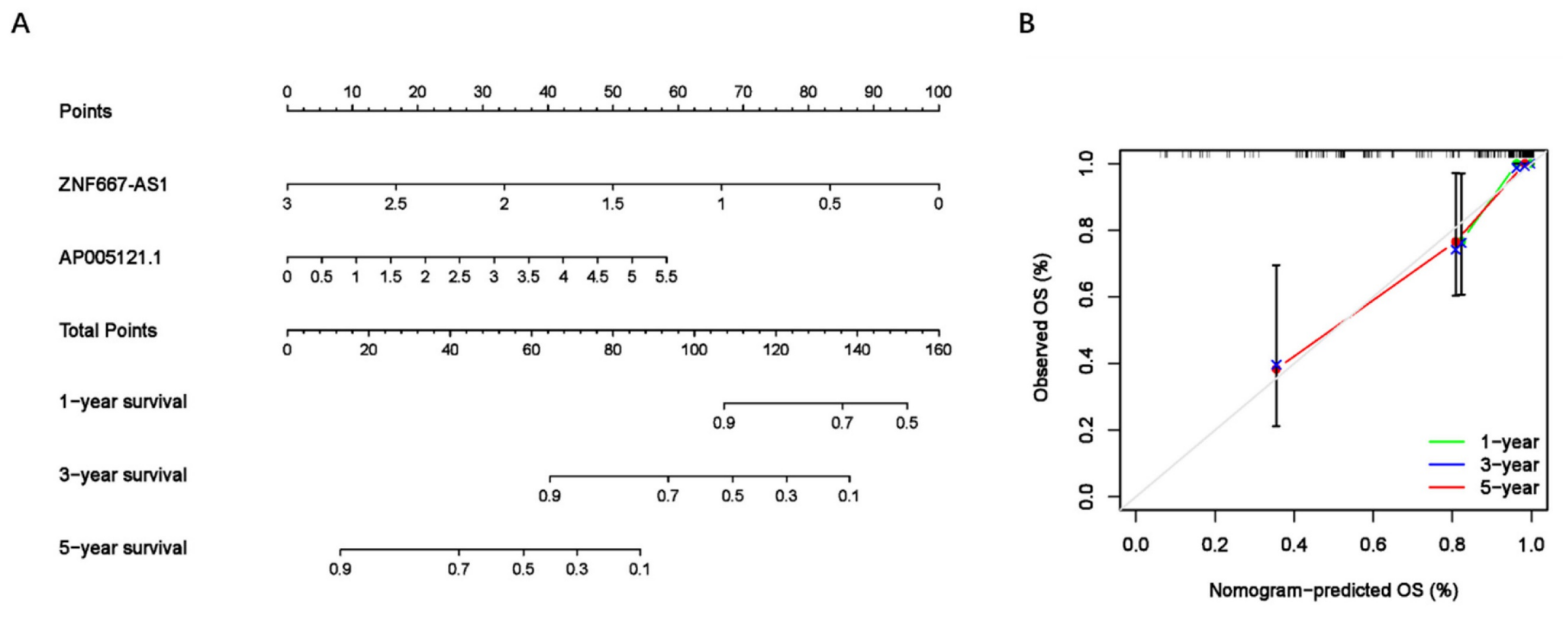
** Nomogram of ER-RSM.** (A) A nomogram is constructed to predict the 1-, 3-, and 5-year survival probability of UM patients by detecting the expression of sER-lncRNAs. (B) The calibration curve of nomogram.

To assess the predictive value of the ER-RSM for UM prognosis, Kaplan-Meier curves demonstrated a significant increase in the risk of death among patients with high-risk scores (Figure 3A). Patients with high expression of ZNF667-AS1 generally had better prognosis compared to those with low expression (Figure 3B), while UM patients with high expression of AP005121.1 had poorer prognosis (Figure 3C). Additionally, ROC curves revealed that the AUC values of the ER-RSM were 0.938, 0.851, and 0.796 for 1-, 3-, and 5-year overall survival, respectively (Figure 4A-4C). Furthermore, the ER-RSM demonstrated higher accuracy in predicting 1-, 3-, and 5-year survival of UM patients compared to other clinical staging systems (gender, stage, T and M staging). Next, we analyzed the relations between the ER-RSM and UM clinical characteristics. We found that ZNF667-AS1 was inversely correlated with higher stage, T, and M staging, while AP005121.1 was positively correlated with higher stage and M staging (Figure 5A-5C). We also conducted independent prognostic factor analysis, which showed that stage and risk scores were significantly related with OS by univariate analysis (P<0.05) (Figure 6A). However, in the multivariate analysis, gender and the risk score demonstrated independent prognostic value (P<0.05) (Figure 6B). Additionally, we incorporated the ER-RSM into nomogram and calibration curves to predict the 1-, 3-, and 5-year survival rates of UM patients (Figure 7A and 7B). Based on the nomogram score, the 1-, 3-, and 5-year survival rates of UM patients could be effectively predicted.

### ER-RSM functional analysis

**Figure 8:**
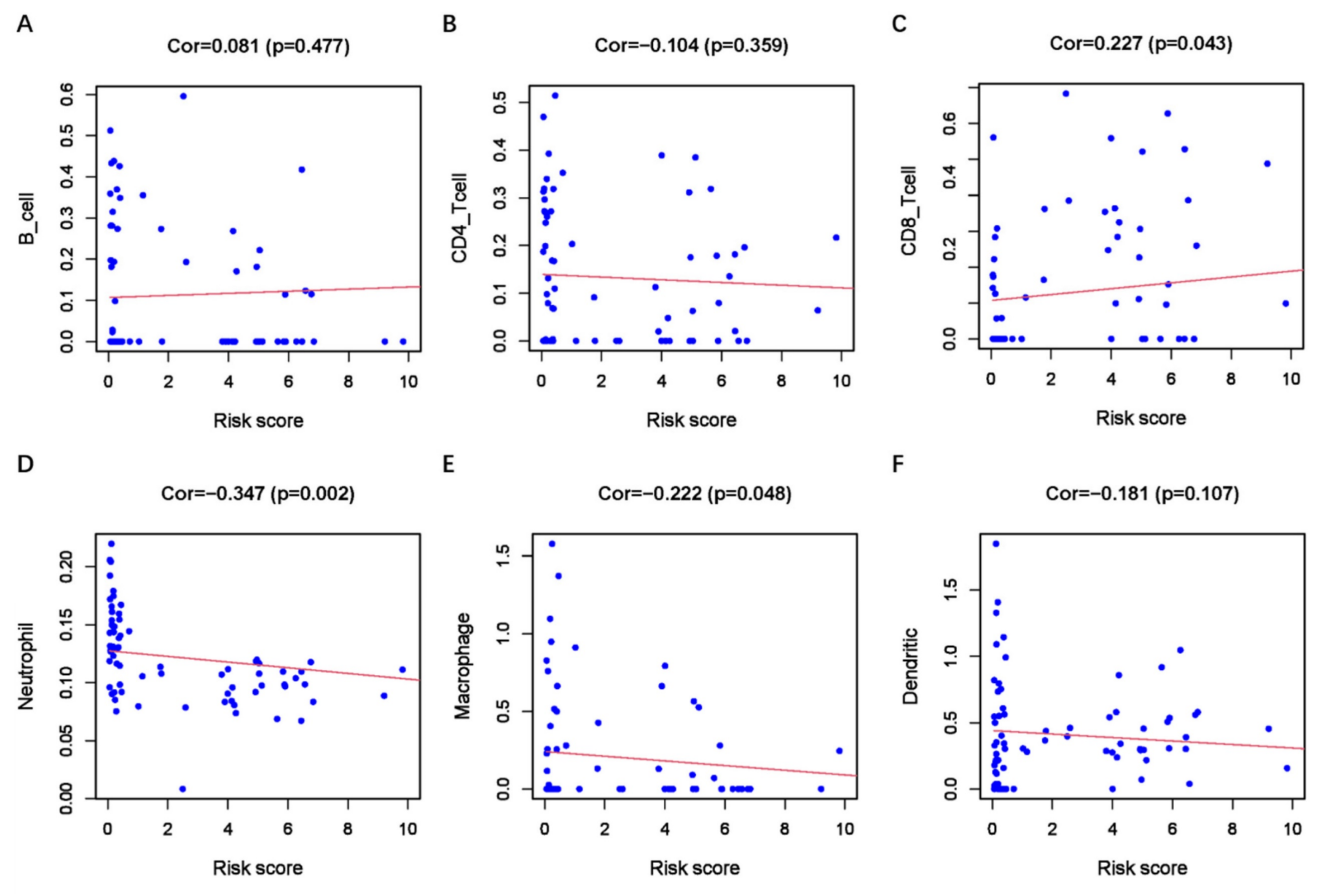
** Relationship between the ER-RSM and infiltration abundances of immune cells.** The relationships between ER-RSM and infiltration abundances of B cells (A); CD4+T cells (B); CD8+T cells (C); neutrophils (D); macrophages (E); dendritic cells (F) are detected by Pearson correlation analysis.

GSEA analysis revealed enrichment of EMT-related Kyoto Encyclopedia of Genes and Genomes (KEGG) pathways. The high-risk group exhibited significant enrichment in EMT-related KEGG pathways, including the TGF-B, WNT, and NOTCH signaling pathways (Supplementary Figure 1A-1C). These results suggest a close association between the ER-RSM and EMT in UM. As immunotherapy emerges as a promising novel treatment for UM, we conducted an analysis of the ER-RSM and immune cell infiltration. We found a significant positive correlation between the ER-RSM and CD8+ T cells, while significant negative correlations were observed with neutrophils and macrophages (Figure 8A-8F).

### The expression of ZNF667-AS1 and AP005121.1 in UM tissues and cell lines

**Figure 9:**
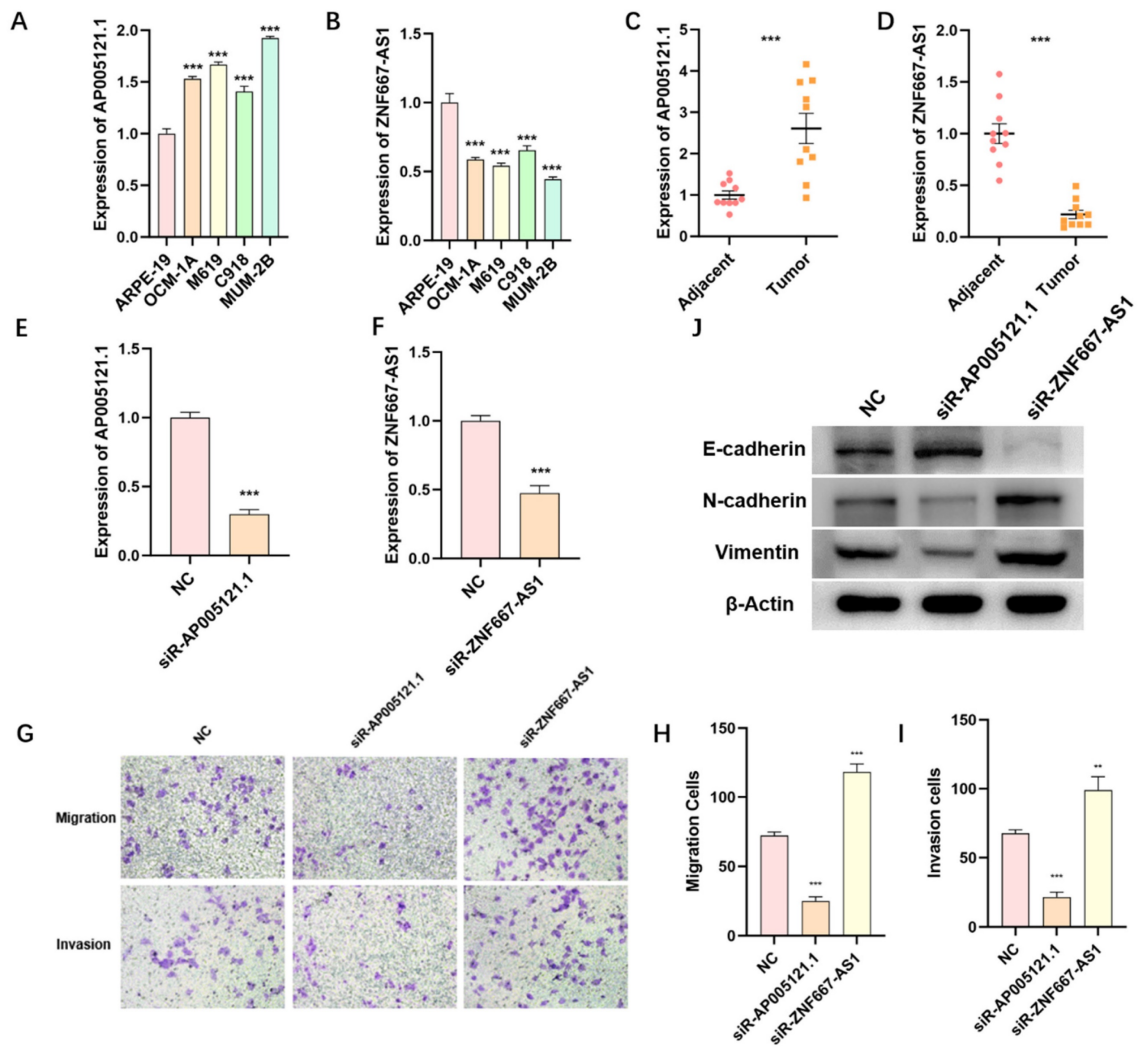
** The expression and function of ZNF667-AS1 and AP005121.1 in UM.** The qPCR results display the expression levels of ZNF667-AS1 (A) and AP005121.1 (B) in UM cell lines and ARPE-19. The expression of ZNF667-AS1 (C) and AP005121.1 (D) in UM tissues and adjacent normal tissues. The knockdown efficiency of ZNF667-AS1 (E) and AP005121.1 (F) is detected by qPCR in MUM-2B cells. (G-I) siR-ZNF667-AS1 promotes the migration and invasion of MUM-2B cells, while siR-AP005121.1 inhibits the migration and invasion of MUM-2B cells. (J) siR-ZNF667-AS1 promotes the expression of Vimentin and N-cadherin, while decreases the expression of E-cadherin; siR-AP005121.1 inhibits the expression of Vimentin and N-cadherin, while promotes the expression of E-cadherin.

We utilized qPCR to detect the expression levels of ZNF667-AS1 and AP005121.1, which were used to establish the ER-RSM, in various UM tissues and cell lines. The expression of AP005121.1 was significantly higher in UM cell lines (OCM-1A, M619, C918, and MUM-2B) compared to retinal pigment epithelial cell line (ARPE-19), whereas ZNF667-AS1 exhibited enhanced expression in ARPE-19 (Figure 9A-9B). We found that AP005121.1 was highly expressed in UM tumor tissues, exhibiting an expression pattern opposite to that of ZNF667-AS1 (Figure 9C-9D).

### The function of ZNF667-AS1 and AP005121.1 in regulating migration and invasion of UM cells

In order to further validate the function of ZNF667-AS1 and AP005121.1 in regulating UM cell migration and invasion, we silenced the expression of ZNF667-AS1 and AP005121.1 using siRNAs in the MUM-2B cell line (Figure 9E-9F). AP005121.1 significantly enhanced the migratory and invasive abilities of UM cells, while ZNF667-AS1 suppressed UM cell migration and invasion (Figure 9G-9I). Furthermore, to verify the impact of siR-ZNF667-AS1 and siR-AP005121.1 on the EMT pathway, we observed that siR-ZNF667-AS1 significantly increased the expression of N-cadherin and Vimentin while reduced the expression of E-cadherin. In contrast, siR-AP005121.1 decreased the expression of N-cadherin and Vimentin while increased the expression of E-cadherin (Figure 9J). These findings indicate that ZNF667-AS1 and AP005121.1 play crucial roles in regulating the EMT process in UM.

## Discussion

Uveal melanoma (UM) is the most common primary intraocular tumor in adults, originating from melanocytes of the choroid, with a strong propensity for metastasis, and the liver being the most common site of metastasis^20^. While optimal methods for treating primary tumors (surgery or radiation therapy) exist, the efficacy for metastatic UM remains unsatisfactory^21^. Therefore, elucidating the mechanisms underlying UM initiation and progression is of paramount importance for early prevention and precise treatment. The 5-year mortality rate for advanced metastatic UM exceeds 95%, underscoring the critical need for new effective prognostic and metastatic biomarkers^22^. Epithelial-mesenchymal transition (EMT) plays a crucial role in cancer pathogenesis and metastasis. EMT is a process by which epithelial cells acquire mesenchymal characteristics and is closely associated with tumor invasion, metastasis, and chemoresistance during tumor progression^23^. Today, the critical role of long non-coding RNAs (lncRNAs) in cancer initiation is widely recognized. Recently, increasing evidence has emphasized the regulatory role of lncRNAs in the EMT process of tumors^24^. Several studies have begun to explore the prognostic value of lncRNAs in cancer from public databases. For instance, Feng *et al.* identified and validated EMT-related lncRNA features to predict survival and immune status in head and neck squamous cell carcinoma^25^; Tao *et al.* identified EMT-related lncRNA features and LINC01116 as an immune-related oncogene in hepatocellular carcinoma^26^; Wang *et al.* discovered vascularization-related lncRNA features to predict the immune microenvironment and prognosis of breast cancer^27^. However, to date, no studies have identified the role of EMT-related lncRNAs in UM. Therefore, there is an urgent need to identify EMT-related lncRNAs in UM, providing novel targets for prognosis prediction and treatment of UM.

In this study, we screened 961 EMT-related lncRNAs and identified 9 sER-lncRNAs (ZNF667-AS1, AC104129.1, L3MBTL4-AS1, AP005121.1, AC005840.2, AC008555.4, AC136475.3, AC124798.1, and AP003390.1) through univariate Cox analysis. Subsequently, an ER-RSM composed of ZNF667-AS1 and AP005121.1 was constructed using multivariate Cox analysis. Patients were classified into high-risk and low-risk groups based on the median risk score. We found that patients in the high-risk group had significantly worse 5-year survival compared to those in the low-risk group, with higher expression of AP003390.1 and lower expression of ZNF667-AS1. Clinical correlation analysis further revealed that the ER-RSM accurately predicted the stage, T, and M staging of UM patients. The AUCs of ROC curves indicated that the ER-RSM effectively predicted the 1-year, 3-year, and 5-year survival of UM patients in different risk groups, and the nomogram further supported the accurate prediction of the 1-year, 3-year, and 5-year survival of UM patients by the ER-RSM. Therefore, the ER-RSM can guide risk stratification and clinical decision-making in UM management. To further validate the expression and function of the ER-RSM through basic experiments, we first detected the expression levels of ZNF667-AS1 and AP005121.1 in UM patient tumor tissues, cell lines, and adjacent tissues. The results showed that AP005121.1 was significantly upregulated in UM tumor tissues and cell lines, while ZNF667-AS1 was significantly downregulated. Additionally, Transwell results demonstrated that ZNF667-AS1 significantly inhibited the migration and invasion abilities of UM cells, while AP005121.1 significantly promoted the migration and invasion abilities of UM cells. Furthermore, siR-ZNF667-AS1 inhibited the EMT pathway (downregulation of E-cadherin protein expression, upregulation of N-cadherin and Vimentin protein expression), while siR-AP005121.1 significantly activated the EMT pathway (upregulation of E-cadherin protein expression, downregulation of N-cadherin and Vimentin protein expression).

Despite revealing the potential of the ER-RSM in prognostic evaluation of UM patients and validating the expression and function of ZNF667-AS1 and AP005121.1 in UM tissues, this study has several limitations: 1) lack of animal experiments for validation; 2) absence of construction of a clinical cohort to validate the ER-RSM.

## Conclusion

In the present study, we uncover the potential of sER-lncRNAs for prognosis evaluation of UM from the EMT and metastasis perspective and reveal their clinical significance. These findings not only help to construct an association between sER-lncRNAs and UM metastasis, but also provide novel targets for future anti-metastasis therapies.

## Supplementary Material

Supplementary figure and table.

## Figures and Tables

**Table 1:** List of primer sequences used in this study

β-actin	F primer	CCTTCCTGGGCATGGAGTC
	R primer	TGATCTTCATTGTGCTGGGTG
ZNF667-AS1	F primer	GGCCCTGCTTTTCATCCTCT
	R primer	CATCCACCCAGTCTCAACCC
AP005121.1	F primer	TCCTTGGATACTCCCTCTGGT
	R primer	AAGGATTTCCAAATTTTCAAATGTGT

Note: F primer: forward primer; R primer: reverse primer.

## Abbreviations

AUC: Area under curve
EMT: Epithelial-mesenchymal transition
ER-lncRNAs: EMT-related lncRNAs
ER-RSM: EMT-related risk score model
GSEA: Gene Set Enrichment Analysis
KEGG: Kyoto Encyclopedia of Genes and Genomes
lncRNAs: Long non-coding RNAs
ROC: Receiver Operating Characteristic
sER-lncRNAs: Survival-related ER-lncRNAs
UM: Uveal melanoma
