# Supplementary material for: A novel EMT-related risk score model for Uveal melanoma based on ZNF667-AS1 and AP005121.1
Source: J Cancer. 2025 Jan 1;16(2):460–9. doi: 10.7150/jca.101823 (PMC11685695; doi:10.7150/jca.101823)
Supplement: Supplementary file 1 — Supplementary figure and table. [file jcav16p0460s1.pdf]

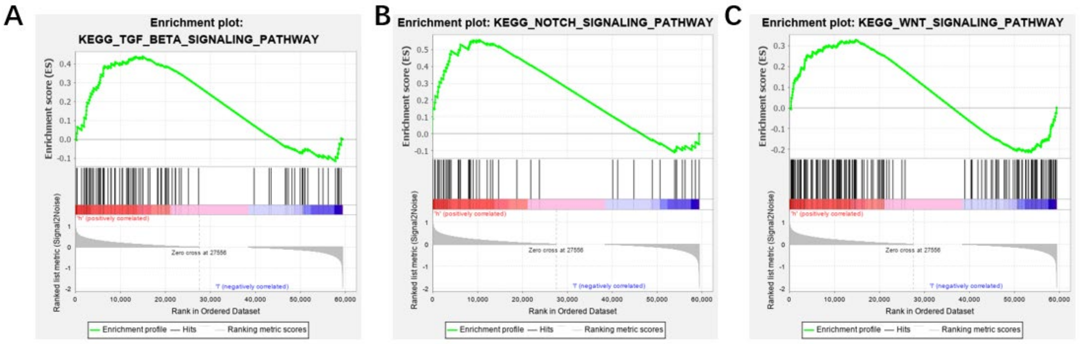

Supplementary Figure 1

KEGG pathway analysis.

The results of KEGG analysis, including (A) TGF-B , (B) WNT, and (C) NOTCH signaling pathways.

Supplementary Table 1: List of siRNAs used in this study

**siR-ZNF667-AS1**	**GAGAGAAGAUGUGAAGUAU(dT)(dT)**
	**AUACUUCACAUCUUCUCUC(dT)(dT)**
**siR-AP005121.1**	**GAUGUCAAGUGUAUAUCUA(dT)(dT)**
	**UAGAUAUACACUUGACAUC(dT)(dT)**
